# The new biology of ageing

**DOI:** 10.1098/rstb.2009.0222

**Published:** 2010-01-12

**Authors:** Linda Partridge

**Affiliations:** Institute of Healthy Ageing and GEE, UCL, Darwin Building, Gower Street, London WC1E 6BT, UK

**Keywords:** ageing, lifespan, ageing-related disease, geriatrics, preventative medicine, nutrient-sensing pathways

## Abstract

Human life expectancy in developed countries has increased steadily for over 150 years, through improvements in public health and lifestyle. More people are hence living long enough to suffer age-related loss of function and disease, and there is a need to improve the health of older people. Ageing is a complex process of damage accumulation, and has been viewed as experimentally and medically intractable. This view has been reinforced by the realization that ageing is a disadvantageous trait that evolves as a side effect of mutation accumulation or a benefit to the young, because of the decline in the force of natural selection at later ages. However, important recent discoveries are that mutations in single genes can extend lifespan of laboratory model organisms and that the mechanisms involved are conserved across large evolutionary distances, including to mammals. These mutations keep the animals functional and pathology-free to later ages, and they can protect against specific ageing-related diseases, including neurodegenerative disease and cancer. Preliminary indications suggest that these new findings from the laboratory may well also apply to humans. Translating these discoveries into medical treatments poses new challenges, including changing clinical thinking towards broad-spectrum, preventative medicine and finding novel routes to drug development.

## 1. Introduction

The increase in life expectancy in human populations worldwide is a triumph of biomedical research. Survival rates started to increase in the mid-nineteenth century, because of improvements in public health, particularly clean water, immunization and antibiotics, and also because of other improvements in lifestyle such as better housing. The rate of increase in life expectancy in most countries does not yet show any sign of slowing and, indeed, is greatest in older age classes; we cannot yet see what any intrinsic limit to human life expectancy will be (Wilmoth 2000; Oeppen & Vaupel 2002).

For a given age, health now is better than it was 150 years ago, but this welcome change is also producing great challenges. Many of these are socio-economic, concerning issues such as work force participation and affordability of pension schemes. Paradoxically, there is also a major medical problem. The improvement in individual health means that larger numbers of individuals reach older ages, and hence live long enough to suffer from ageing-related disease and loss of function. All of the major killer diseases, including cardiovascular disease, cancer and dementia, are strongly age related. The predominant burden of ill-health is now falling on the older section of the population and, both for health benefits to ageing individuals and economic benefits to the societies in which they live, we urgently need to discover means of improving health during ageing. Fortunately, major scientific opportunities have opened up in research into ageing and bring with them the enticing prospect of a broad-spectrum, preventative, medicine for diseases of ageing. However, taking the fruits of these scientific discoveries to the ageing human population may not be straightforward.

From the biological standpoint, the major features of ageing are an intrinsic decline in function during adulthood, leading to a drop in fecundity and increased likelihood of death (Finch 1990). Ageing is not inevitable and, indeed, some organisms seem not to age at all or to do so very slowly. Some even show an increase in fecundity or survival rate over at least part of adulthood. Ageing is particularly apparent in organisms where growth is completed before reproduction commences, such as insects, birds and many mammals, including humans (Vaupel *et al*. 2004; Baudisch 2005). The major laboratory model organisms used for research into ageing, namely budding yeast *Saccharomyces cerevisiae*, the nematode worm *Caenorhabditis elegans*, the fruitfly *Drosophila melanogaster* and the mouse *Mus musculus*, all fall into this category and, in this sense at least, are good models for human ageing.

The phenotypes associated with ageing have been best studied in humans and are complex (Martin 2002). Within single tissues, multiple types of damage and pathology increase in incidence with age, and the spectrum of changes differs between tissues. The precise phenotypes of ageing are also notably variable between individuals (Finch & Kirkwood 1999). This complexity and variability have led to a picture of the ageing process as intractable, for both experimental analysis and medical intervention. Indeed, it could be concluded that there is no single ageing process; rather, during ageing, a large number of independent and stochastic processes of damage accumulation occur in parallel, with little or no common causality. Amelioration of the impact of one type of ageing-related damage would, if this scenario is correct, leave the majority unaffected and would hence have little impact on overall ageing-related decline. This view of ageing permeates medicine to the present day. Geriatrics is largely a primary care medical speciality, with little input from basic and clinical research, unlike specific ageing-related diseases such as cancer, cardiovascular disease and neurodegeneration, which are all associated with sizeable and well-funded research communities. Specific diseases of ageing are generally viewed as medically tractable, unlike the ageing process itself.

The idea that ageing is difficult to modify has until recently been reinforced by work on its evolution. Evolutionary biologists have long been intrigued by ageing, because it is a deleterious trait, but it nonetheless shows great diversity in the natural world. After various ideas of a possible benefit of ageing to family groups or whole species were largely discredited (Kirkwood & Cremer 1982), the key insight came with the realization that, because of extrinsic causes of mortality such as disease, predation and accidents, the force of natural selection weakens for older age classes, because fewer individuals succeed in reaching them (Haldane 1941; Medawar 1946, 1952). A substantial body of theoretical analysis, experimentation and comparative work led to the conclusion that ageing can hence evolve as a side effect, either of pressure of new mutations that reduce fecundity or survival probability later in life or of mutations that have beneficial effects in the young (Medawar 1952; Williams 1957; Hamilton 1966; Hughes & Reynolds 2005; Partridge & Gems 2006; Moorad & Promislow 2008). As far as we know, no genes have evolved to cause ageing. Unlike development, there is no well-oiled hierarchy of genetic regulation to ensure that ageing happens in the right tissues and at the right times. Instead, it is an unregulated side effect of the failure of natural selection to maintain function at the later ages that few individuals reach in nature (Partridge & Gems 2002*a*). These theoretical and practical insights have led to the conclusion that ageing is likely to be a highly polygenic trait, since many genes are involved in assurance of survival during adulthood and in promoting fecundity.

The complexity of the ageing phenotype and the realization that it is an evolutionary side effect, rather than an adaptive process, led to the widespread assumption that mutations in single genes were unlikely to be capable of slowing down ageing. Furthermore, it seemed improbable that mechanisms of ageing would be the same in different kinds of organisms. If different human tissues acquire such different forms of damage and pathology during ageing, presumably as a result of the different types of insults of daily living that they encounter then, by the same token, organisms with very different life styles would be expected to encounter different sources of damage (Partridge & Gems 2002*b*).

## 2. Single-gene mutations that extend the lifespan of laboratory animals

Perhaps the single most important advance in ageing research in recent years has been discovery of mutations in single genes that extend the lifespan of laboratory animals. They first came to light as a result of a systematic chemical mutagenesis screen for lifespan-extending mutations in *C. elegans* (Klass 1983). Subsequent work with these mutations (Friedman & Johnson 1988), and further screening (Kenyon *et al*. 1993), revealed that it was possible to double the lifespan of the worm with a mutation in a single gene. Furthermore, rather than solely prolonging the moribund period at the end of the life, the mutations caused the worms to remain healthy and youthful for longer (Kenyon *et al*. 1993). The mutated genes were discovered to encode components of an invertebrate insulin/insulin-like growth-factor-like signalling (IIS) pathway (Kimura *et al*. 1997; Lin *et al*. 1997; Ogg *et al*. 1997). These findings came as a considerable surprise, because a signalling pathway previously associated with control of growth and metabolism in mammals now turned out to play a role in determination of lifespan in a distantly related invertebrate.

Mutations with similar effects on lifespan were soon discovered in other model organisms. For instance, a similar screening effort in yeast led to the discovery that over-expression of a protein deacetylase, *SIR2*, extended replicative lifespan (Sinclair & Guarente 1997; Kaeberlein *et al.* 1999), while mutations in *methuselah* in *Drosophila* increased fly lifespan (Lin Seroude & Benzer 1998). Likewise, in the mouse, mutations in genes encoding transcription factors involved in the development of the pituitary gland resulted in long-lived dwarf mice (Brown-Borg *et al*. 1996). By the late 1990s, it was firmly established that lifespan of these model organisms could indeed be extended by mutations in single genes.

It had also been known since the 1930s that an environmental intervention, dietary restriction (DR), could produce substantial increases in lifespan in laboratory rodents (McCay *et al.* 1935). Although the exact mechanisms at work still await full elucidation, detailed study of DR rodents has demonstrated a broad-spectrum improvement in health and a delay in or amelioration of the impact of a wide range of ageing-related diseases (Masoro 2005, 2006). For instance, the animals are protected against cancer, cataract, diabetes, motor decline, osteoporosis and nephropathy (Weindruch & Walford 1988). These findings suggested that, in principle, multiple aspects of the ageing phenotype could be simultaneously ameliorated by a single intervention, albeit, in the case of DR, a complex one.

## 3. Evolutionary conservation

The ultimate aim of biomedical research into ageing with animals is to improve the health of the older section of human populations. Laboratory model organisms have been key to understanding many other aspects of human biology. Embryonic development, the cell cycle, the functioning of the nervous system, cellular metabolism and many other processes have often been investigated by proceeding from simpler organisms to more complex ones. This process works because of evolutionary conservation of genes and their functions over the large evolutionary distances involved. Indeed, it is often possible to introduce a human gene into yeast or *Drosophila* and find that it functions quite normally there. However, because ageing is not an adaptive trait and because different kinds of organisms are exposed to different kinds of stress and damage, there has been a good reason to doubt that this kind of evolutionary conservation will apply to the ageing process.

DR extends lifespan not only in rodents but also in a wide range of distantly related organisms, including yeast (Jiang *et al*. 2000; Lin *et al.* 2000), *C. elegans* (Klass 1977; Lakowski & Hekimi 1998; Greer *et al*. 2007; Kennedy *et al.* 2007; Smith *et al*. 2008*a*) and *Drosophila* (Chippindale *et al*. 1993; Chapman & Partridge 1996). Indeed recent work has demonstrated that DR increases lifespan in rhesus monkeys (Holloszy & Fontana 2007; Mattison *et al*. 2007; Colman *et al*. 2009) and short-term DR can produce improvements in function in humans (e.g. Holloszy & Fontana 2007). Because the details of the mechanisms by which DR extends lifespan are not fully elucidated for any organism, it is not clear whether this is a case of evolutionary conservation or whether instead there has been evolutionary convergence (Mair & Dillin 2008).

It was originally suspected that extension of lifespan by reduced IIS might turn out to be a worm peculiarity. This was because mutations in genes in the IIS pathway can also cause the worms to enter a type of developmental arrest (dauer), normally seen only in response to low food or crowding (Riddle & Albert 1997). Dauer larvae are long lived, and the long life of IIS mutant adult worms could therefore have been a result of re-expression in the adult of the genes that make the dauer larva long lived (Kenyon *et al*. 1993), a speculation confirmed by studies of gene expression (McElwee *et al.* 2003, 2004). Most organisms do not undergo this type of developmental arrest and might therefore lack the mechanisms for long life seen in dauer larvae. However, an important recent discovery has been that the IIS pathway has an evolutionarily conserved role in determining longevity; mechanisms of ageing therefore are, at least to some extent, ‘public’ or shared (Partridge & Gems 2002*b*). Remarkably, mutations in the single *Drosophila* insulin receptor (Tatar *et al*. 2001) and insulin receptor substrate (Clancy *et al*. 2001) proved to extend lifespan in the fly. Furthermore, mutations in the genes encoding both the insulin (Bluher *et al.* 2003) and Igf-1 receptor (Holzenberger *et al*. 2003) extended lifespan in the mouse. Subsequent work with all three organisms has amply confirmed the evolutionarily conserved role of this signalling pathway (Russell & Kahn 2007; Piper *et al*. 2008; Taguchi & White 2008). Early evidence from population–genetic association studies has also started to implicate the pathway in determination of human lifespan (Mooijaart *et al*. 2005; Kuningas *et al*. 2007; Willcox *et al*. 2008).

Evidence for evolutionary conservation of genetic determinants of lifespan is at present strongest for the IIS pathway, but others are likely to lengthen the list. For instance, the effect of elevated expression of *SIR2* in yeast appears to be conserved in *C. elegans* (Tissenbaum & Guarente 2001) and *Drosophila* (Rogina & Helfand 2004), and mutations in genes encoding components of the target of rapamycin (TOR) pathway also extend the lifespan in all four organisms (Jia *et al.* 2004; Kapahi *et al*. 2004; Kaeberlein *et al*. 2005; Hansen *et al*. 2007; Pan *et al*. 2007; Sheaffer *et al.* 2008; Smith *et al*. 2008*b*; Harrison *et al*. 2009). Sufficient single-gene mutations that extend lifespan in yeast and *C. elegans* have now been identified to allow a quantitative estimate of the degree of evolutionary conservation of genetic modifiers of ageing between these two organisms (Smith *et al*. 2008*b*). In *C. elegans*, loss of function of a set of approximately 276 genes, or altered function of their protein products, has proved to extend lifespan. A set of 103 yeast orthologues of 78 of these 276 worm genes could be identified on the basis of sequence similarity, and deletion of 76 of these resulted in viable yeast strains. Eleven of the 76 were long lived, a proportion 4.3 times higher than would be expected from deletion of the same number of randomly selected yeast genes. Many of the genes with a conserved role in ageing in these two organisms are involved in protein synthesis (Smith *et al*. 2008*b*), a process whose importance to ageing has recently been demonstrated by experimental studies in *C. elegans* (Hansen *et al*. 2007; Pan *et al*. 2007). This strong signal of evolutionary conservation between these two distantly related organisms suggests that future studies of the role of protein synthesis in ageing in the fruitfly and the mouse would pay dividends.

Although there is abundant evidence for an evolutionarily conserved role for IIS and other pathways in determination of lifespan, it remains to be seen how deep that conservation penetrates. Even at the level of signalling mechanisms, there may be considerable variation between different organisms as is implied, for instance, by the presence of much larger numbers of insulin ligands in the worm (38) and the fly (7) than in mammals. In addition, similar changes in signalling in different organisms may have very different outcomes because of differences in structure and physiology. Of particular importance for IIS, insulin resistance and failure in insulin production can result in diabetes in mammals, with its consequent vascular damage, while the invertebrates, with their open circulatory systems, can probably better tolerate elevated blood sugar. Only a narrow range of alterations in IIS may therefore increase mammalian lifespan. There is some evidence for evolutionary conservation of the biochemical mechanisms by which altered IIS extends lifespan in different organisms. For instance, profiling of gene expression in long-lived, IIS mutant worms, flies and mice showed increased expression of genes encoding components of phase 1 and 2 detoxification pathway, important in the elimination of lipophilic endobiotics, xenobiotics and drugs (McElwee *et al*. 2007; Sykiotis & Bohmann 2008; Tullet *et al*. 2008). Subsequent work with a key transcriptional regulator of the pathway has demonstrated experimentally that increasing its activity can increase lifespan in both *C. elegans* and *Drosophila* (McElwee *et al*. 2007; Sykiotis & Bohmann 2008; Tullet *et al*. 2008). Cellular detoxification may therefore be an important process for protection against the effects of ageing in all three organisms, although whether the toxins involved are the same or different remains to be determined.

So far we have only scratched the surface of the mechanisms at work in lifespan extension. Nonetheless, these new findings have opened up the promise of a major scientific opportunity, to use the invertebrates and the mouse to understand human ageing, exploiting the full range of analytical tools available in the model organisms.

## 4. Risk and damage

Slowing down ageing is not the only means by which lifespan can be extended. The ageing process is characterized by a decline in function with advancing age during adulthood; the state of the organisms progressively worsens. One might therefore expect that an intervention that extended lifespan by amelioration of the ageing process would do so by slowing down the rate at which state worsens with age (Finch 1990). A simple and direct way of assessing the state of a population is to measure mortality rate, which is, to a first approximation, the proportion of individuals that enter each age class that die during it. Mortality rates generally show a roughly exponential increase with age in humans and the laboratory model organisms and can hence be described in terms of two important parameters: the initial, baseline mortality rate, which is age independent, and the rate at which mortality rate increases with age (Finch 1990; Pletcher *et al*. 2000). Interventions, genetic and environmental, that increase lifespan can do so by decreasing either or both of these parameters (Pletcher *et al*. 2000). A reduction in the slope of a mortality trajectory is what would be expected if lifespan were increased by a reduction in the rate of ageing itself (Finch 1990).

One intervention that clearly can slow down the rate of ageing is lowered temperature for ectotherms. In *Drosophila*, lowered temperature increases lifespan entirely by lowering the slope of the mortality trajectory, with no effect on the initial mortality rate (Mair *et al*. 2003). These flies are too small to thermoregulate and are thus forced to adopt ambient temperature. Lowering of the slope of the mortality trajectory in cooler environments is consistent with the idea that lowered temperature decreases the rate of most or all molecular processes in the organism, including the rate of ageing. In support of this view, when flies are switched between temperatures, the subsequent slope of the mortality trajectory immediately changes to that characteristic of flies kept permanently in the new thermal regime (Mair *et al*. 2003). The flies therefore bear the permanent imprint of their thermal history, with warmer temperatures leading to the accumulation of a higher level of irreversible damage, and no acute effect of temperature on mortality rate. Lowered temperature thus decreases the rate of ageing in *Drosophila* and provides a useful benchmark for an intervention that does so.

Rather than decreasing the rate of ageing, the increase in lifespan in industrialized human societies has occurred by a reduction in baseline mortality rates, with no reduction in the slope of the mortality trajectory (Wilmoth 2000). This suggests that overall health, at all ages, has improved, but that the underlying process of accumulation of ageing-related damage has not been ameliorated. This finding leaves open the question of the time course of these effects. For instance, events early in life or even *in utero* could have a lifelong impact on health, and there could also be more acute effects of recent and current environments. To measure such timing effects, it is necessary to compare individuals with currently similar circumstances but different past environments, and *vice versa*.

Interestingly, DR can have a similar effect on mortality trajectories to that associated with the increase in human lifespan expectancy; DR extends life in *Drosophila* entirely by reducing the initial mortality rate with no lowering of its slope (Pletcher *et al*. 2000). Similar findings have been reported for DR in mice (Weindruch *et al*. 1986; Hursting *et al.* 1994), and for one form of DR in *C. elegans* (Smith *et al*. 2008*a*), suggesting that, in these three organisms at least, DR may not slow down the rate of ageing and may instead increase lifespan through a different mechanism. Indeed, experimental reversal of the nutritional status of flies has shown that the effect of DR on mortality rate is acute. Later onset DR leads, within 48 h, to a switch in subsequent mortality rates to those of permanently DR flies (Mair *et al*. 2003). Likewise, previously DR flies that are switched to full feeding at later ages show a rapid increase in mortality rates to those characteristic of flies that are permanently fully fed. DR and fully fed flies thus age at the same rate, and DR instead extends lifespan by reducing the acute risk of death.

There is little information on the timing of the effects of single-gene mutations on mortality rate. In *C. elegans*, switches in IIS status using double stranded RNA interference have shown that the pathway acts specifically during adulthood to determine adult survival (Dillin Crawford & Kenyon 2002), but more detailed timing information is not yet available. In *Drosophila*, an inducible system for gene expression was used to show that, at least up to a month of adult age, the IIS pathway acts acutely to determine mortality rate, similar to DR (Giannakou *et al*. 2007). It will be important to determine whether this kind of acute effect on mortality rate applies to other pathways that determine lifespan and, in particular, whether it extends to mammals. But it is already clear that, in principle, lifespan can be extended by making the animal less likely to die of the damage that it has accumulated, rather than by reducing the accumulation of damage.

## 5. Ageing and ageing-related diseases

It has long been known that DR in rodents reduces the impact of a wide range of ageing-related diseases, and it has also been shown to reduce the impact of proteotoxicity in *C. elegans* (Steinkraus *et al*. 2008). Because the single-gene mutations that extend lifespan have only been discovered recently, less information is available, but already it seems that aspects of function and health during ageing are improved. For instance, associative learning is more strongly maintained at later ages in long-lived IIS mutants worms (Murakami *et al*. 2005), while locomotor function is better maintained during ageing in long-lived IIS mutant flies (Martin & Grotewiel 2006). Loss of the insulin receptor substrate 1 in the mouse also protects against loss of glucose homeostasis, immune and motor function and reduces the impact of osteoporosis, cataract and ulcerative dermatitis (Selman *et al*. 2008). As well as maintaining function and health during ageing, lifespan-extending mutations can protect against the pathology associated with specific genetic models of ageing-related disease. For instance, recent work with *C. elegans* has revealed that mutations in IIS that increase lifespan can reduce the pathology associated with genetic models of cancer (Pinkston *et al*. 2006; Pinkston-Gosse & Kenyon 2007) and of proteotoxicity-induced neurodegeneration (Cohen *et al*. 2006; Pinkston *et al*. 2006; Steinkraus *et al*. 2008). Furthermore, mutations in IIS in the mouse can protect against the pathology associated with specific genetic models of Alzheimer's disease (Freude *et al*. 2009; Killick *et al*. 2009). The indications are, therefore, that these interventions can produce an improvement in health and function in diverse tissue systems and reduce the impact of ageing-related diseases with diverse aetiology (Butler *et al*. 2008).

The implication of these findings is that protection against the ageing process results in protection against diverse, ageing-related diseases. The ageing process itself is acting as the major risk factor for these conditions. This realization leads in turn to the conclusion that there is an underlying commonality in the aetiology of these ageing-related diseases, despite their diverse manifestations. It is early days yet, and a great deal more work needs to be done to understand exactly how the ageing process increases vulnerability to these diseases. We need also to understand how transition into loss of function and disease occurs, and how a single environmental intervention or gene mutation can have such broad-spectrum effects. A key challenge in the biology of ageing, and one that is increasingly being recognized, is to understand how events at different levels of organization contribute to loss of function during organismal ageing and to eventual death (Kirkwood 2008; Murphy & Partridge 2008). Presumably, in a complex chain of events, damage to macromolecules and organelles causes decline in cellular function and cell loss, which in turn compromise the function of tissues. Dysfunctional tissues could in turn act systemically to cause stress and eventual damage to other tissues, which could to some extend cause a correlation in the rate of ageing of different parts of the body within an individual. Many of these key changes may be susceptible to acute intervention, similar to the effects of DR in the invertebrate model organisms. At some point, irreversibility must enter the system, because of the emergence of lethal, ageing-related disease that cannot be rescued by the intervention (Partridge Pletcher & Mair 2005*b*). Identifying, experimentally investigating and modelling these temporal changes and their dynamics will require considerable effort, and in the near future much more experimental work will be needed to bring understanding of these systems to a level of maturity where productive modelling will be possible.

## 6. Will lifespan extension in laboratory model organisms be relevant to human ageing?

The findings from the model organisms have a clear, potential message for the medical treatment of ageing-related diseases (Butler *et al*. 2008). At present, these diseases are treated piecemeal by different medical specialists, because they are regarded as separate medical problems. Patients themselves generally visit a clinician because they have a specific medical problem, not because they are old. However, if in humans, also, protection against the effect of ageing can delay or ameliorate diverse ageing-related diseases, then a quite different approach to the health of older people would pay dividends. A broad-spectrum, preventative approach would be required, with individuals who reached a certain age being treated even in the absence of any ageing-related disease. Furthermore, if the effects of a beneficial intervention were acute, as has occurred in some of the animal models, then it would need to be applied for the rest of life. Clinical trials would also need to be conducted for a protracted period. All of these features would pose significant obstacles to translating the findings from basic science into drug development and clinical practice. However, if the findings from the animal models turn out to apply to humans, then a major opportunity could be missed. What, then, is the likelihood that evolutionary conservation of the mechanisms will extend to our own species?

There are some obvious questions about lifespan extension in animal models that have a bearing on likely relevance to humans. If these single gene mutations can produce such broad-spectrum benefits to health, then why is the mutant not the wild-type? These mutants must have side effects that mean that they are not the fittest genotype in the wild. Some mutants that extend lifespan clearly delay or reduce fecundity, as does DR (Partridge *et al.* 2005*a*). However, it is also clear that, at least in the laboratory, impaired fecundity is not necessary for extension of lifespan by some single-gene mutations (Partridge *et al*. 2005*a*), although some claims that this is the case may have been based on failing to measure all aspects of fecundity or doing so in benign circumstances (Rogina *et al*. 2000; Walker *et al*. 2000; Marden *et al*. 2003; Jenkins *et al.* 2004). Nature is in general a more exacting place than the laboratory, where the animals are kept largely free of pathogens, have an abundant and highly accessible food supply and are kept largely free of competition with conspecifics. However, many of these considerations apply also to humans in developed countries. It will be important to evaluate what are the negative effects of single-gene mutations that make them disadvantageous under natural circumstances, to understand how important these might be for humans. It should also be borne in mind that medical interventions into ageing are likely to be applied only later in the lifespan, when some of the negative side effects may no longer be relevant, and it has already been demonstrated that administration of a TOR inhibitor, rapamycin, later in life in mice can extend the lifespan (Harrison *et al*. 2009). The prospects that the findings from the laboratory will prove to be of medical relevance to humans therefore look promising.

Humans are, obviously, much longer lived than any of the laboratory model organisms. This could have a bearing on the extent to which interventions could ameliorate the effects of ageing, or not. It is notable that many of the genes that have so far come to light as affecting longevity in the laboratory are involved in nutrient sensing pathways, which contribute to matching the growth and reproductive rate of the animals to their nutritional status. Human growth and reproduction respond to nutrients, but not to such an extent as do those of the laboratory model organisms, which are all subject to boom and bust conditions in nature. However, even if human lifespan is not as plastic as that of laboratory animals, the same may not be true for ageing-related disease. The aim of this research is to improve human health during ageing, not to extend lifespan *per se*, and it remains to be seen to what extent this is going to be possible.

## References

[RSTB20090222C1] Baudisch A. 2005 Hamilton's indicators of the force of selection. Proc. Natl Acad. Sci. USA 102, 8263–8268 (doi:10.1073/pnas.0502155102) 15919822 10.1073/pnas.0502155102 PMC1140481

[RSTB20090222C2] Bluher M. Kahn B. Kahn C. 2003 Extended longevity in mice lacking the insulin receptor in adipose tissue. Science 299, 572–574 (doi:10.1126/science.1078223) 12543978 10.1126/science.1078223

[RSTB20090222C3] Brown-Borg H. M. Borg K. E. Meliska C. J. Bartke A. 1996 Dwarf mice and the ageing process. Nature 384, 33 (doi:10.1038/384033a0) 8900272 10.1038/384033a0

[RSTB20090222C4] Butler R. N. 2008 New model of health promotion and disease prevention for the 21st century. BMJ 337, a399 (doi:10.1136/bmj.a399) 18614506 10.1136/bmj.a399 PMC2483908

[RSTB20090222C5] Chapman T. Partridge L. 1996 Female fitness in *Drosophila melanogaster*: an interaction between the effect of nutrition and of encounter rate with males. Proc. Biol. Sci. 263, 755–759 (doi:10.1098/rspb.1996.0113) 8763795 10.1098/rspb.1996.0113

[RSTB20090222C6] Chippindale A. K. Leroi A. Kim S. B. Rose M. R. 1993 Phenotypic plasticity and selection in *Drosophila* life history evolution. 1. Nutrition and the cost of reproduction. J. Evol. Biol. 6, 171–193 (doi:10.1046/j.1420-9101.1993.6020171.x)

[RSTB20090222C7] Clancy D. J. Gems D. Harshman L. G. Oldham S. Stocker H. Hafen E. Leevers S. J. Partridge L. 2001 Extension of life-span by loss of CHICO, a *Drosophila* insulin receptor substrate protein. Science 292, 104–106 (doi:10.1126/science.1057991) 11292874 10.1126/science.1057991

[RSTB20090222C8] Cohen E. Bieschke J. Perciavalle R. M. Kelly J. W. Dillin A. 2006 Opposing activities protect against age-onset proteotoxicity. Science 313, 1604–1610 (doi:10.1126/science.1124646) 16902091 10.1126/science.1124646

[RSTB20090222C9] Colman R. J. 2009 Caloric restriction delays disease onset and mortality in rhesus monkeys. Science 325, 201–204 (doi:10.1126/science.1173635) 19590001 10.1126/science.1173635 PMC2812811

[RSTB20090222C10] Dillin A. Crawford D. K. Kenyon C. 2002 Timing requirements for insulin/IGF-1 signaling in *C. elegans*. Science 298, 830–834 (doi:10.1126/science.1074240) 12399591 10.1126/science.1074240

[RSTB20090222C11] Finch C. E. 1990 Longevity, senescence and the genome Chicago, IL: University of Chicago Press

[RSTB20090222C12] Finch C. E. Kirkwood T. 1999 Chance, development and ageing Oxford, UK: Oxford University Press

[RSTB20090222C13] Freude S. 2009 Neuronal IGF-1 resistance reduces A*β* accumulation and protects against premature death in a model of Alzheimer's disease. FASEB J. 23, 3315–3324 (doi:10.1096/fj.09-132043) 19487308 10.1096/fj.09-132043

[RSTB20090222C14] Friedman D. B. Johnson T. E. 1988 Three mutants that extend both mean and maximum life span of the nematode, *Caenorhabditis elegans*, define the *age-1* gene. J. Gerontol. Biol. Sci. 43, B102–B109 10.1093/geronj/43.4.b102 3385139

[RSTB20090222C15] Giannakou M. E. Goss M. Jacobson J. Vinti G. Leevers S. J. Partridge L. 2007 Dynamics of the action of dFOXO on adult mortality in *Drosophila*. Aging Cell 6, 429–438 (doi:10.1111/j.1474-9726.2007.00290.x) 17465980 10.1111/j.1474-9726.2007.00290.x

[RSTB20090222C16] Greer E. L. Dowlatshahi D. Banko M. R. Villen J. Hoang K. Blanchard D. Gygi S. P. Brunet A. 2007 An AMPK-FOXO pathway mediates longevity induced by a novel method of dietary restriction in *C. elegans*. Curr. Biol. 17, 1646–1656 (doi:10.1016/j.cub.2007.08.047) 17900900 10.1016/j.cub.2007.08.047 PMC2185793

[RSTB20090222C17] Haldane J. B. S. 1941 New paths in genetics London, UK: Allen and Unwin

[RSTB20090222C18] Hamilton W. D. 1966 The moulding of senescence by natural selection. J. Theor. Biol. 12, 12–45 (doi:10.1016/0022-5193(66)90184-6) 6015424 10.1016/0022-5193(66)90184-6

[RSTB20090222C19] Hansen M. Taubert S. Crawford D. Libina N. Lee S. J. Kenyon C. 2007 Lifespan extension by conditions that inhibit translation in *Caenorhabditis elegans*. Aging Cell 6, 95–110 (doi:10.1111/j.1474-9726.2006.00267.x) 17266679 10.1111/j.1474-9726.2006.00267.x

[RSTB20090222C20] Harrison D. E. 2009 Rapamycin fed late in life extends lifespan in genetically heterogeneous mice. Nature 460, 392–395 19587680 10.1038/nature08221 PMC2786175

[RSTB20090222C21] Holloszy J. O. Fontana L. 2007 Caloric restriction in humans. Exp. Gerontol. 42, 709–712 (doi:10.1016/j.exger.2007.03.009) 17482403 10.1016/j.exger.2007.03.009 PMC2020845

[RSTB20090222C22] Holzenberger M. Dupont J. Ducos B. Leneuve P. Geloen A. Even P. C. Cervera P. Le Bouc Y. 2003 IGF-1 receptor regulates lifespan and resistance to oxidative stress in mice. Nature 421, 182–187 (doi:10.1038/nature01298) 12483226 10.1038/nature01298

[RSTB20090222C23] Hughes K. A. Reynolds R. M. 2005 Evolutionary and mechanistic theories of aging. Annu. Rev. Entomol. 50, 421–445 (doi:10.1146/annurev.ento.50.071803.130409) 15355246 10.1146/annurev.ento.50.071803.130409

[RSTB20090222C24] Hursting S. D. Perkins S. N. Phang J. M. 1994 Calorie restriction delays spontaneous tumorigenesis in p53-knockout transgenic mice. Proc. Natl Acad. Sci. USA 91, 7036–7040 (doi:10.1073/pnas.91.15.7036) 8041741 10.1073/pnas.91.15.7036 PMC44333

[RSTB20090222C25] Jenkins N. L. McColl G. Lithgow G. J. 2004 Fitness cost of extended lifespan in *Caenorhabditis elegans*. Proc. R. Soc. Lond. B 271, 2523–2526 (doi:10.1098/rspb.2004.2897) 10.1098/rspb.2004.2897 PMC1440519 15590605

[RSTB20090222C26] Jia K. Chen D. Riddle D. 2004 The TOR pathway interacts with the insulin signaling pathway to regulate *C. elegans* larval development, metabolism and life span. Development 131, 3897–3906 (doi:10.1242/dev.01255) 15253933 10.1242/dev.01255

[RSTB20090222C27] Jiang J. C. Jaruga E. Repnevskaya M. V. Jazwinski S. M. 2000 An intervention resembling caloric restriction prolongs life span and retards aging in yeast. FASEB J 14, 2135–2137 11024000 10.1096/fj.00-0242fje

[RSTB20090222C28] Kaeberlein M. McVey M. Guarente L. 1999 The *SIR2/3/4* complex and *SIR2* alone promote longevity in *Saccharomyces cerevisiae* by two different mechanisms. Genes Dev. 13, 2570–2580 (doi:10.1101/gad.13.19.2570) 10521401 10.1101/gad.13.19.2570 PMC317077

[RSTB20090222C29] Kaeberlein M. 2005 Regulation of yeast replicative life span by TOR and Sch9 in response to nutrients. Science 310, 1193–1196 (doi:10.1126/science.1115535) 16293764 10.1126/science.1115535

[RSTB20090222C30] Kapahi P. Zid B. M. Harper T. Koslover D. Sapin V. Benzer S. 2004 Regulation of lifespan in *Drosophila* by modulation of genes in the TOR signaling pathway. Curr. Biol. 14, 885–890 (doi:10.1016/j.cub.2004.03.059) 15186745 10.1016/j.cub.2004.03.059 PMC2754830

[RSTB20090222C31] Kennedy B. K. Steffen K. K. Kaeberlein M. 2007 Ruminations on dietary restriction and aging. Cell Mol. Life Sci. 64, 1323–1328 (doi:10.1007/s00018-007-6470-y) 17396225 10.1007/s00018-007-6470-y PMC11136230

[RSTB20090222C32] Kenyon C. Chang J. Gensch E. Rudener A. Tabtiang R. 1993 A *C. elegans* mutant that lives twice as long as wild type. Nature 366, 461–464 (doi:10.1038/366461a0) 8247153 10.1038/366461a0

[RSTB20090222C34] Killick R. 2009 Deletion of Irs2 reduces amyloid deposition and rescues behavioural deficits in APP transgenic mice. Biochem. Biophys. Res. Commun. 386, 257–262 (doi:10.1016/j.bbrc.2009.06.032) 19523444 10.1016/j.bbrc.2009.06.032 PMC2726921

[RSTB20090222C35] Kimura K. D. Tissenbaum H. A. Liu Y. Ruvkun G. 1997 *daf-2*, an insulin receptor-like gene that regulates longevity and diapause in *Caenorhabditis elegans*. Science 277, 942–946 (doi:10.1126/science.277.5328.942) 9252323 10.1126/science.277.5328.942

[RSTB20090222C36] Kirkwood T. B. 2008 A systematic look at an old problem. Nature 451, 644–647 (doi:10.1038/451644a) 18256658 10.1038/451644a

[RSTB20090222C37] Kirkwood T. B. L. Cremer T. 1982 Cytogerontology since 1881: a reappraisal of August Weismann and a review of modern progress. Hum. Genet. 60, 101–121 (doi:10.1007/BF00569695) 7042533 10.1007/BF00569695

[RSTB20090222C38] Klass M. R. 1977 Aging in the nematode *Caenorhabditis elegans*: major biological and environmental factors influencing life span. Mech. Ageing Dev. 6, 413–429 (doi:10.1016/0047-6374(77)90043-4) 926867 10.1016/0047-6374(77)90043-4

[RSTB20090222C39] Klass M. R. 1983 A method for the isolation of longevity mutants in the nematode *Caenorhabditis elegans* and initial results. Mech. Ageing Dev. 22, 279–286 (doi:10.1016/0047-6374(83)90082-9) 6632998 10.1016/0047-6374(83)90082-9

[RSTB20090222C40] Kuningas M. Magi R. Westendorp R. G. Slagboom P. E. Remm M. van Heemst D. 2007 Haplotypes in the human *Foxo1a* and *Foxo3a* genes; impact on disease and mortality at old age. Eur. J. Hum. Genet. 15, 294–301 (doi:10.1038/sj.ejhg.5201766) 17245409 10.1038/sj.ejhg.5201766

[RSTB20090222C41] Lakowski B. Hekimi S. 1998 The genetics of caloric restriction in *Caenorhabditis elegans*. Proc. Natl. Acad. Sci. USA 95, 13091–13096 (doi:10.1073/pnas.95.22.13091) 9789046 10.1073/pnas.95.22.13091 PMC23719

[RSTB20090222C42] Lin K. Dorman J. B. Rodan A. Kenyon C. 1997 *daf-16*: An HNF-3/forkhead family member that can function to double the life-span of *Caenorhabditis elegans*. Science 278, 1319–1322 (doi:10.1126/science.278.5341.1319) 9360933 10.1126/science.278.5341.1319

[RSTB20090222C43] Lin Y. J. Seroude L. Benzer S. 1998 Extended life-span and stress resistance in the *Drosophila* mutant methuselah. Science 282, 943–946 (doi:10.1126/science.282.5390.943) 9794765 10.1126/science.282.5390.943

[RSTB20090222C44] Lin S. J. Defossez P. A. Guarente L. 2000 Requirement of NAD and SIR2 for life-span extension by calorie restriction in *Saccharomyces cerevisiae*. Science 289, 2126–2128 (doi:10.1126/science.289.5487.2126) 11000115 10.1126/science.289.5487.2126

[RSTB20090222C45] Mair W. Dillin A. 2008 Aging and survival: the genetics of life span extension by dietary restriction. Annu. Rev. Biochem. 77, 727–754 (doi:10.1146/annurev.biochem.77.061206.171059) 18373439 10.1146/annurev.biochem.77.061206.171059

[RSTB20090222C46] Mair W. Goymer P. Pletcher S. Partridge L. 2003 Demography of dietary restriction and death in *Drosophila*. Science 301, 1731–1733 (doi:10.1126/science.1086016) 14500985 10.1126/science.1086016

[RSTB20090222C47] Marden J. H. Rogina B. Montooth K. L. Helfand S. L. 2003 Conditional tradeoffs between aging and organismal performance of Indy long-lived mutant flies. Proc. Natl Acad. Sci. USA 100, 3369–3373 (doi:10.1073/pnas.0634985100) 12626742 10.1073/pnas.0634985100 PMC152299

[RSTB20090222C48] Martin G. M. 2002 Keynote: mechanisms of senescence–complificationists versus simplificationists. Mech. Ageing Dev. 123, 65–73, discussion 75–79 (doi:10.1016/S0047-6374(01)00335-9) 11718800 10.1016/s0047-6374(01)00335-9

[RSTB20090222C49] Martin I. Grotewiel M. S. 2006 Distinct genetic influences on locomotor senescence in *Drosophila* revealed by a series of metrical analyses. Exp. Gerontol. 41, 877–881 (doi:10.1016/j.exger.2006.06.052) 16891076 10.1016/j.exger.2006.06.052

[RSTB20090222C50] Masoro E. J. 2005 Overview of caloric restriction and ageing. Mech. Ageing Dev. 126, 913–922 (doi:10.1016/j.mad.2005.03.012) 15885745 10.1016/j.mad.2005.03.012

[RSTB20090222C51] Masoro E. J. 2006 Caloric restriction and aging: controversial issues. J. Gerontol. A Biol. Sci. Med. Sci. 61, 14–19 16456190 10.1093/gerona/61.1.14

[RSTB20090222C52] Mattison J. A. Roth G. S. Lane M. A. Ingram D. K. 2007 Dietary restriction in aging nonhuman primates. Interdiscipl. Top. Gerontol. 35, 137–158 10.1159/000096560 17063037

[RSTB20090222C53] McCay C. M. Crowell M. F. Maynard L. A. 1935 The effect of retarded growth upon the length of lifespan and upon the ultimate body size. J. Nutr. 10, 63–79 2520283

[RSTB20090222C54] McElwee J. Bubb K. Thomas J. 2003 Transcriptional outputs of the *Caenorhabditis elegans* forkhead protein DAF-16. Aging Cell 2, 111–121 (doi:10.1046/j.1474-9728.2003.00043.x) 12882324 10.1046/j.1474-9728.2003.00043.x

[RSTB20090222C55] McElwee J. J. Schuster E. Blanc E. Thomas J. H. Gems D. 2004 Shared transcriptional signature in *C. elegans* dauer larvae and long-lived *daf-2* mutants implicates detoxification system in longevity assurance. J. Biol. Chem. 279, 44533–44543 (doi:10.1074/jbc.M406207200) 15308663 10.1074/jbc.M406207200

[RSTB20090222C56] McElwee J. J. 2007 Evolutionary conservation of regulated longevity assurance mechanisms. Genome Biol. 8, R132 (doi:10.1186/gb-2007-8-7-r132) 17612391 10.1186/gb-2007-8-7-r132 PMC2323215

[RSTB20090222C57] Medawar P. B. 1946 Old age and natural death. Mod. Q. 2, 30–49

[RSTB20090222C58] Medawar P. B. 1952 An unsolved problem of biology London, UK: H.K. Lewis

[RSTB20090222C59] Mooijaart S. P. 2005 *C. elegans* DAF-12, nuclear hormone receptors and human longevity and disease at old age. Ageing Res. Rev. 4, 351–371 (doi:10.1016/j.arr.2005.03.006) 16051528 10.1016/j.arr.2005.03.006

[RSTB20090222C60] Moorad J. A. Promislow D. E. 2008 A theory of age-dependent mutation and senescence. Genetics 179, 2061–2073 (doi:10.1534/genetics.108.088526) 18660535 10.1534/genetics.108.088526 PMC2516080

[RSTB20090222C61] Murakami H. Bessinger K. Hellmann J. Murakami S. 2005 Aging-dependent and -independent modulation of associative learning behavior by insulin/insulin-like growth factor-1 signal in *Caenorhabditis elegans*. J. Neurosci. 25, 10894–10904 (doi:10.1523/JNEUROSCI.3600-04.2005) 16306402 10.1523/JNEUROSCI.3600-04.2005 PMC6725869

[RSTB20090222C62] Murphy M. P. Partridge L. 2008 Toward a control theory analysis of aging. Annu. Rev. Biochem. 77, 777–798 (doi:10.1146/annurev.biochem.77.070606.101605) 18318658 10.1146/annurev.biochem.77.070606.101605 PMC4335186

[RSTB20090222C63] Oeppen J. Vaupel J. W. 2002 Demography. Broken limits to life expectancy. Science 296, 1029–1031 (doi:10.1126/science.1069675) 12004104 10.1126/science.1069675

[RSTB20090222C64] Ogg S. Paradis S. Gottlieb S. Patterson G. I. Lee L. Tissenbaum H. A. Ruvkun G. 1997 The fork head transcription factor DAF-16 transduces insulin-like metabolic and longevity signals in *C. elegans*. Nature 389, 994–999 9353126 10.1038/40194

[RSTB20090222C65] Pan K. Z. Palter J. E. Rogers A. N. Olsen A. Chen D. Lithgow G. J. Kapahi P. 2007 Inhibition of mRNA translation extends lifespan in *Caenorhabditis elegans*. Aging Cell 6, 111–119 (doi:10.1111/j.1474-9726.2006.00266.x) 17266680 10.1111/j.1474-9726.2006.00266.x PMC2745345

[RSTB20090222C66] Partridge L. Gems D. 2002a A lethal side-effect. Nature 418, 921 (doi:10.1038/418921a) 12198525 10.1038/418921a

[RSTB20090222C67] Partridge L. Gems D. 2002b Mechanisms of ageing: public or private? Nat. Rev. Genet. 3, 165–175 (doi:10.1038/nrg753) 11972154 10.1038/nrg753

[RSTB20090222C68] Partridge L. Gems D. 2006 Beyond the evolutionary theory of ageing, from functional genomics to evo-gero. Trends Ecol. Evol. 21, 334–340 (doi:10.1016/j.tree.2006.02.008) 16769434 10.1016/j.tree.2006.02.008

[RSTB20090222C69] Partridge L. Gems D. Withers D. J. 2005a Sex and death: what is the connection? Cell 120, 461–472 (doi:10.1016/j.cell.2005.01.026) 15734679 10.1016/j.cell.2005.01.026

[RSTB20090222C70] Partridge L. Pletcher S. D. Mair W. 2005b Dietary restriction, mortality trajectories, risk and damage. Mech. Ageing Dev. 126, 35–41 (doi:10.1016/j.mad.2004.09.017) 15610760 10.1016/j.mad.2004.09.017

[RSTB20090222C71] Pinkston J. M. Garigan D. Hansen M. Kenyon C. 2006 Mutations that increase the life span of *C. elegans* inhibit tumor growth. Science 313, 971–975 (doi:10.1126/science.1121908) 16917064 10.1126/science.1121908

[RSTB20090222C72] Pinkston-Gosse J. Kenyon C. 2007 DAF-16/FOXO targets genes that regulate tumor growth in *Caenorhabditis elegans*. Nat. Genet. 39, 1403–1409 (doi:10.1038/ng.2007.1) 17934462 10.1038/ng.2007.1

[RSTB20090222C73] Piper M. D. Selman C. McElwee J. J. Partridge L. 2008 Separating cause from effect: how does insulin/IGF signalling control lifespan in worms, flies and mice? J. Intern. Med. 263, 179–191 18226095 10.1111/j.1365-2796.2007.01906.x

[RSTB20090222C74] Pletcher S. Khazaeli A. A. Curtsinger J. W. 2000 Why do life spans differ? Partitioning mean longevity differences in terms of age-specific mortality parameters. J. Gerontol. A Biol. Sci. Med. Sci. 55, B381–B389 10952359 10.1093/gerona/55.8.b381

[RSTB20090222C75] Riddle D. L. Albert P. S. 1997 Genetic and environmental regulation of dauer larva development. In C. elegans II (eds Riddle D. L. Blumenthal T. Meyer B. J. Priess J. R.), pp. 739–768 Plainview, NY: Cold Spring Harbor Laboratory Press 21413222

[RSTB20090222C76] Rogina B. Helfand S. L. 2004 Sir2 mediates longevity in the fly through a pathway related to calorie restriction. Proc. Natl Acad. Sci. USA 101, 15998–16003 (doi:10.1073/pnas.0404184101) 15520384 10.1073/pnas.0404184101 PMC528752

[RSTB20090222C77] Rogina B. Reenan R. A. Nilsen S. P. Helfand S. L. 2000 Extended life-span conferred by cotransporter gene mutations in *Drosophila*. Science 290, 2137–2140 (doi:10.1126/science.290.5499.2137) 11118146 10.1126/science.290.5499.2137

[RSTB20090222C78] Russell S. J. Kahn C. R. 2007 Endocrine regulation of ageing. Nat. Rev. Mol. Cell Biol. 8, 681–691 (doi:10.1038/nrm2234) 17684529 10.1038/nrm2234

[RSTB20090222C79] Selman C. 2008 Evidence for lifespan extension and delayed age-related biomarkers in insulin receptor substrate 1 null mice. FASEB J. 22, 807–818 (doi:10.1096/fj.07-9261com) 17928362 10.1096/fj.07-9261com

[RSTB20090222C80] Sheaffer K. L. Updike D. L. Mango S. E. 2008 The target of rapamycin pathway antagonizes pha-4/FoxA to control development and aging. Curr. Biol. 18, 1355–1364 (doi:10.1016/j.cub.2008.07.097) 18804378 10.1016/j.cub.2008.07.097 PMC2615410

[RSTB20090222C81] Sinclair D. Guarente L. 1997 Extrachromosomal rDNA circles—a cause of aging in yeast. Cell 91, 1033–1042 (doi:10.1016/S0092-8674(00)80493-6) 9428525 10.1016/s0092-8674(00)80493-6

[RSTB20090222C82] Smith E. D. Kaeberlein T. L. Lydum B. T. Sager J. Welton K. L. Kennedy B. K. Kaeberlein M. 2008a Age-and calorie-independent life span extension from dietary restriction by bacterial deprivation in *Caenorhabditis elegans*. BMC Dev. Biol. 8, 49 (doi:10.1186/1471-213X-8-49) 18457595 10.1186/1471-213X-8-49 PMC2408926

[RSTB20090222C83] Smith E. D. 2008b Quantitative evidence for conserved longevity pathways between divergent eukaryotic species. Genome Res. 18, 564–570 (doi:10.1101/gr.074724.107) 18340043 10.1101/gr.074724.107 PMC2279244

[RSTB20090222C84] Steinkraus K. A. Smith E. D. Davis C. Carr D. Pendergrass W. R. Sutphin G. L. Kennedy B. K. Kaeberlein M. 2008 Dietary restriction suppresses proteotoxicity and enhances longevity by an hsf-1-dependent mechanism in *Caenorhabditis elegans*. Aging Cell 7, 394–404 (doi:10.1111/j.1474-9726.2008.00385.x) 18331616 10.1111/j.1474-9726.2008.00385.x PMC2709959

[RSTB20090222C85] Sykiotis G. P. Bohmann D. 2008 Keap1/Nrf2 signaling regulates oxidative stress tolerance and lifespan in *Drosophila*. Dev. Cell 14, 76–85 (doi:10.1016/j.devcel.2007.12.002) 18194654 10.1016/j.devcel.2007.12.002 PMC2257869

[RSTB20090222C86] Taguchi A. White M. F. 2008 Insulin-like signaling, nutrient homeostasis, and life span. Annu. Rev. Physiol. 70, 191–212 (doi:10.1146/annurev.physiol.70.113006.100533) 17988211 10.1146/annurev.physiol.70.113006.100533

[RSTB20090222C87] Tatar M. Kopelman A. Epstein D. Tu M. P. Yin C. M. Garofalo R. S. 2001 A mutant *Drosophila* insulin receptor homolog that extends life-span and impairs neuroendocrine function. Science 292, 107–110 (doi:10.1126/science.1057987) 11292875 10.1126/science.1057987

[RSTB20090222C88] Tissenbaum H. A. Guarente L. 2001 Increased dosage of a *sir-2* gene extends life span in *Caenorhabditis elegans*. Nature 410, 227–230 (doi:10.1038/35065638) 11242085 10.1038/35065638

[RSTB20090222C89] Tullet J. M. Hertweck M. An J. H. Baker J. Hwang J. Y. Liu S. Oliveira R. P. Baumeister R. Blackwell T. K. 2008 Direct inhibition of the longevity-promoting factor SKN-1 by insulin-like signaling in *C. elegans*. Cell 132, 1025–1038 (doi:10.1016/j.cell.2008.01.030) 18358814 10.1016/j.cell.2008.01.030 PMC2367249

[RSTB20090222C90] Vaupel J. W. Baudisch A. Dolling M. Roach D. A. Gampe J. 2004 The case for negative senescence. Theor. Popul. Biol. 65, 339–351 15136009 10.1016/j.tpb.2003.12.003

[RSTB20090222C91] Walker D. W. McColl G. Jenkins N. L. Harris J. Lithgow G. J. 2000 Evolution of lifespan in *C. elegans*. Nature 405, 296–297 10830948 10.1038/35012693

[RSTB20090222C92] Weindruch R. Walford R. 1988 The retardation of aging and disease by dietary restriction Springfiled, IL: Charles C Thomas

[RSTB20090222C93] Weindruch R. Walford R. Fligiel S. Guthrie D. 1986 The retardation of aging in mice by dietary restriction: longevity, cancer, immunity and lifetime energy intake. J. Nutr. 116, 641–654 3958810 10.1093/jn/116.4.641

[RSTB20090222C94] Willcox B. J. 2008 FOXO3A genotype is strongly associated with human longevity. Proc. Natl Acad. Sci. USA 105, 13987–13992 (doi:10.1073/pnas.0801030105) 18765803 10.1073/pnas.0801030105 PMC2544566

[RSTB20090222C95] Williams G. C. 1957 Pleiotropy, natural selection and the evolution of senescence. Evolution 11, 398–411 (doi:10.2307/2406060)

[RSTB20090222C96] Wilmoth J. 2000 Demography of longevity: past, present, and future trends. Exp. Gerontol. 35, 1111–1129 (doi:10.1016/S0531-5565(00)00194-7) 11113596 10.1016/s0531-5565(00)00194-7

